# Giant cell-rich osteosarcoma of the lumbar spine – A rare entity

**DOI:** 10.4102/sajr.v28i1.2839

**Published:** 2024-05-09

**Authors:** Tushar Kalekar, Ojasvi Sharma, Sayali Paidlewar, Ankita Pandey, Eshan C. Durgi

**Affiliations:** 1 Department of Radiodiagnosis, Dr. DY Patil Medical College, Hospital and Research Centre, Pune, India

**Keywords:** osteosarcoma, giant cell, lumbar spine, lumbo-sacral, spinal tumour

## Abstract

Giant cell-rich osteosarcoma (GCRO) is a rare variant of osteosarcoma. A significant diagnostic hurdle is presented by the lesion’s uncommon appearance on histopathology. This report presents a case of a 59-year-old female patient diagnosed with GCRO.

**Contribution:**

While it is extremely difficult to distinguish GCRO from malignant giant cell tumour, it is important to do so because of the difference in prognosis and management. Distinctive anatomy of the lumbar spine increases the risks associated with surgical excision.

## Introduction

The most prevalent non-haematologic primary malignancy affecting the bone is osteosarcoma.^1^ It is characterised by the production of disorganised osteoid tissue from mesenchymal cancer cells.^2^ Giant cell-rich osteosarcoma (GCRO) is an uncommon variant of osteosarcoma initially reported in 1986 by Bathurst et al.,^3^ accounting for only 1% – 3% of all conventional osteosarcomas.^2,4^ According to the 2020 classification of bone tumours by the World Health Organization, GCRO is classified as conventional osteosarcoma, falling within the category of osteosarcoma not otherwise specified.^5^ Giant cell-rich osteosarcoma is an undifferentiated high-grade sarcoma that exhibits varying amounts of tumour osteoid with an abundance of osteoclast-like giant cells.^6^

Spinal osteosarcomas are uncommon, comprising 3.6% – 14.5% of primary tumours of the spine and 0.85% – 3.0% of all osteosarcomas.^7^ In contrast to long-bone GCRO, spinal GCRO is incredibly rare and poses unique challenges for its diagnosis and management.^8^ Because of the difficulty in differentiating between its clinical, radiological and histopathological features, osteosarcoma of the spine is frequently initially misinterpreted as an aggressive benign vertebral lesion like osteoblastoma or aneurysmal bone cyst and can sometimes can be mistaken for chondrosarcoma or malignant giant cell tumour (GCT).^1,9^ A biopsy is therefore usually indicated. The atypical GCRO variant is characterised by an abundance of osteoclast-like giant cells and paucity of osteoid tumour,^3^ resulting in confusion with GCTs.^10,11^ It has been reported that a Ki67 proliferative index of 20% – 30% infiltration of surrounding trabeculae and focal osteoid deposits are helpful for distinguishing it from GCT.^10^

## Case report

A 59-year-old woman presented to the orthopaedic department in April 2023 with a chief complaint of backache radiating to the right lower limb for 2 years, associated with weakness and tingling in the right lower limb and difficulty walking. There was no history of preceding fever, co-morbidities or relevant surgery. On examination, swelling was noted in the lower lumbar region associated with tenderness.

**FIGURE 1:**
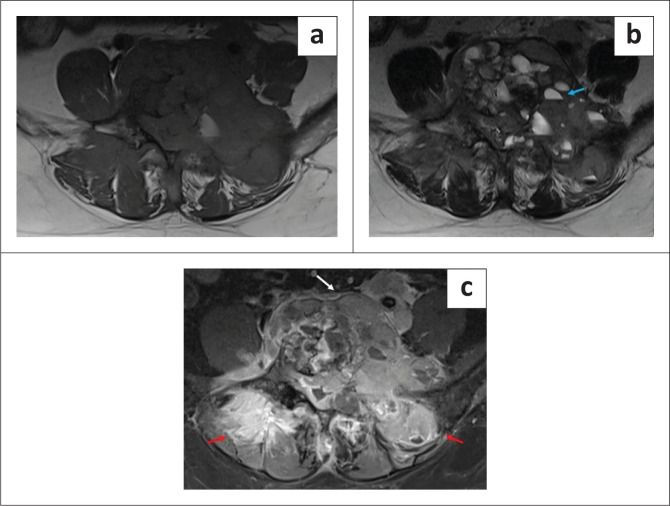
MRI axial T1-weighted (T1WI), T2-weighted (T2WI) and post-contrast T1 Fat Saturated sequence (T1FS). (a–c) demonstrate an expansile lesion of altered signal intensity involving the body of the L5 vertebra, appearing hypointense on T1WI and T2WI with marked post-contrast enhancement. There is involvement of the left transverse process, left lamina and superior articular processes of the L5 vertebra. (b) The lesion shows multiple fluid-fluid levels with a supernatant hyperintense component and a dependent hypointense component suggestive of haemorrhage (blue arrow). (c) An associated large enhancing soft tissue component is seen involving the prevertebral and paravertebral regions and bilateral erector spinae muscles (red arrow). Anteriorly, the mass is partially encasing the common iliac vessels (white arrow).

**FIGURE 2:**
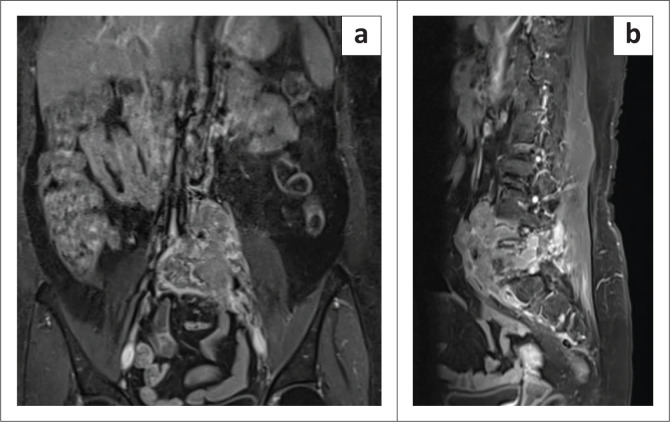
MRI coronal and sagittal post-contrast T1 Fat Saturated sequence (T1FS) images. The lesion shows marked post-contrast enhancement and is seen encasing the aortic bifurcation and left common iliac vessels (a). Sagittal image showing the cranio-caudal extension of the lesion from the anterior aspect of the inferior endplate of the L3 vertebra to the anterior aspect of the S1 vertebral body (b).

Prior pre- and post-contrast MRI of the lumbo-sacral spine was acquired in January 2023 using a Siemens Magnetom Vida 3T MRI. The study revealed a destructive L5 vertebral body lesion with complete marrow replacement, hypointense on T1 and T2WI with multiple foci of hyperintensity on the Short Tau Inversion Recovery (STIR) sequence. T2W and post-contrast T1FS axial images revealed a large, lobulated soft tissue mass arising from the body of the L5 vertebra and extending into the left anterolateral and posterolateral paraspinal soft tissues. The posterolateral component demonstrated multiple fluid levels on T2WI with a hyperintense supernatant component and a hypointense dependent component, likely representing haemorrhage. The left transverse process, left lamina and both superior articular processes of the L5 vertebra were also involved. The lesion extended intraspinally with encasement of the thecal sac at the L4-L5 and L5-S1 levels resulting in marked compression of the thecal sac. Severe compromise of the L4-L5 and L5-S1 lateral recesses and neural foramina was noted with significant stenosis resulting in compression of both L4 and L5 exiting nerve roots and L5 and S1 traversing nerve roots. Anteriorly, the lesion was abutting the aortic bifurcation and encasing the left iliac vessels, as well as compressing the left lower ureter with ipsilateral hydroureteronephrosis. On the right, there was extension to the right pedicle, right superior articular process and medial portion of the right transverse process of the L5 vertebra (Figure 1 and 2). The MRI findings were suggestive of an aggressive vertebral lesion showing multiple fluid levels, suggestive of GCT and osteosarcoma.^12^

A PET-CT was performed at an external hospital in April 2023. In comparison to the previous MRI, the PET-CT revealed a significant interval increase in the bulk, extent of involvement and metabolic activity of the lesion in and around the L5 vertebra. There was progressive involvement of the left iliacus, bilateral psoas and erector spinae muscles. A few necrotic fluorodeoxyglucose (FDG) avid retroperitoneal and iliac lymph nodes were seen.

**FIGURE 3:**
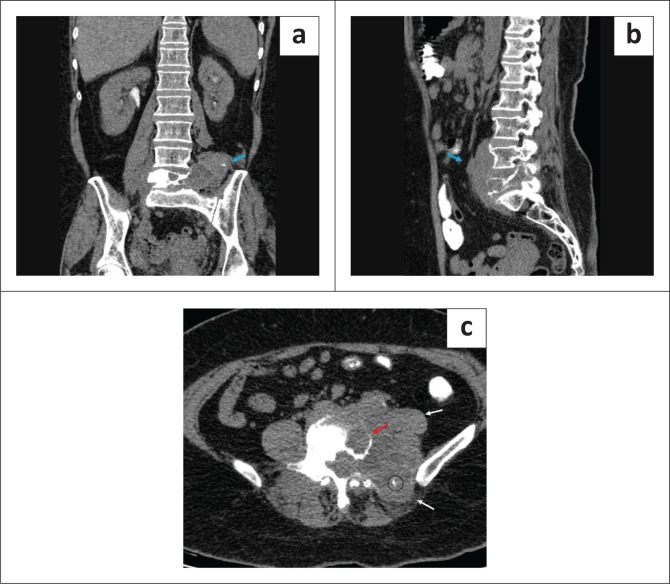
Non-contrast CT scan. An ill-defined soft tissue density lesion is seen along the anterior aspect of the inferior endplate of the L3 vertebra to the anterior aspect of the S1 vertebral body and extending laterally from the L5 vertebral body to the right and left paravertebral regions, predominantly on the left side (blue arrow) (a–b). The L5 vertebral body appears sclerotic and shows an expansile destructive lytic lesion involving the vertebral body with complete destruction of the left lateral vertebral body, left transverse process, left pedicle, left lamina of vertebral arch and left superior articular process (red arrow). Posteriorly, the lesion is seen extending between the left iliac crest and left transverse process of the L4 vertebra and is seen compressing the left posterior paraspinal musculature. Anteriorly, the lesion is seen displacing the left psoas muscle laterally (white arrows). A few foci of calcification are seen within the soft tissue mass (circle) (c).

Lumbar spine CT was also performed in April 2023 using the Philips ingenuity core 128-slice multidetector computed tomography (MDCT) scanner. The study revealed an expansile, lytic lesion in the L5 vertebral body with complete destruction of the left transverse process, left pedicle, left lamina and left superior articular process (Figure 3). Tiny lytic areas were also seen involving the right pedicle, right transverse process, right lamina and right superior articular process.

A repeat MRI of the lumbo-sacral spine was acquired at the same time. Compared with the previous MRI, the vertebral destruction showed no significant change; however, the peridural extension of the mass and left posterolateral soft tissue component showed definite worsening.

**FIGURE 4:**
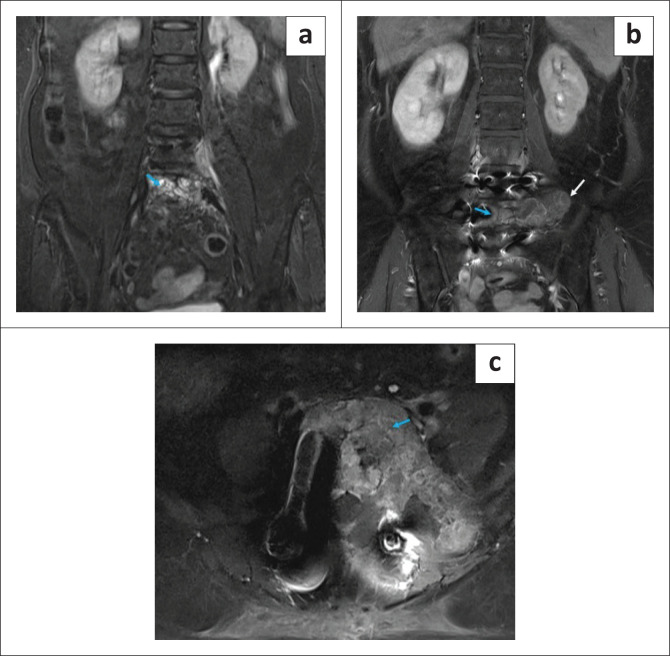
Post-operative MRI Short Tau Inversion Recovery (STIR) coronal, post-contrast T1 Fat Saturated sequence (T1FS) coronal and axial images. (a–c) A persistent expansile lesion is seen involving the body of the L5 vertebra (blue arrow). The lesion shows multiple hyperintense foci on the STIR sequence (a) and heterogeneous post-contrast enhancement on the T1FS sequence (b, c). (b) Residual soft tissue involvement is mainly on the left side (white arrow).

The patient underwent a L4-L5 laminectomy with decompression of the thecal sac in April 2023, with a perceived improvement of symptoms following surgical intervention. A post-operative MRI scan was performed after 1 month. There was a significant reduction of the tumour, as well as in the associated enhancing epidural component and the soft tissue extension in the prevertebral and paravertebral regions (Figure 4). Marked reduction of the adjacent nerve root compression was also seen at this level. Residual soft tissue involvement was predominantly noted involving the left neural foramen with resultant compromise of the left exiting nerve roots. The residual lesion involving the paraspinal soft tissue was seen extending along the left psoas, erector spinae and iliacus muscles. Marked reduction in the size and number of lymph nodes was seen. There was also a marked reduction in the extrinsic compression of the left ureter with no evidence of hydroureteronephrosis.

The histopathology report described the tumour as sheets of mononuclear cells having round to oval nuclei and a moderate amount of cytoplasm with numerous multinucleate giant cells and eosinophilic osteoid-like material within the tumour substance. The findings were representative of a giant cell-rich lesion.

## Discussion

Giant cell-rich osteosarcoma is an uncommon variant of osteosarcoma. It may easily be incorrectly identified as a malignant GCT of the bone, both radiographically and histologically. Ilaslan et al. calculated that in the axial skeleton, the incidence of primary osteosarcoma was merely 4%, based on a study of 4887 osteosarcoma cases.^1^ Bathurst et al. reported that GCRO is primarily found in long bones and makes up about 3% of all osteosarcoma cases.^3^

Ilaslan et al. studied 198 cases of primary spinal osteosarcoma, including 95 male and 103 female patients, ranging in age from 8 to 80 years, with an average age of 34.5 years. There were 27 cases (13.6%) of the cervical vertebrae, 66 (33.3%) of the thoracic vertebrae, 64 (32.3%) of the lumbar vertebrae and 41 of the sacral vertebrae (20.7%).^1^

The World Health Organization classified osteosarcoma histologically into surface, intramedullary and central variants with several subtypes. There are four subtypes of central osteosarcoma – low-grade osteosarcoma, small-cell osteosarcoma, telangiectatic osteosarcoma and conventional osteosarcoma. Osteoblastic, chondroblastic and fibroblastic variants are relatively common.^13,14^

In eukaryotes, the structure of the nucleosome is determined by basic nuclear proteins called histones. A mutation of H3K27 me3, which affects the trimethylated lysine residue at position 27 in the protein histone H3, may be seen in GCRO.^15^ Amplification of the murine double minute 2 (MDM2) and cyclin dependent kinase 4 (CDK4) genes has also been reported to be the cause of GCRO, which usually develops from a prior low-grade osteosarcoma.^15^

Compared to other vertebral tumours, osteosarcoma frequently affects several continuous vertebral bodies.^16^ Imaging findings of GCRO are similar to those of GCT of the bone.^17^ Also, due to the similarities in histopathological features between GCRO and GCT, GCRO may be frequently misdiagnosed as GCT.^18^ The common radiographic features shared by GCT and GCRO are radiolucency on X-ray images and osteolytic lesions on CT.^18,19^ The primary characteristic feature on histopathology that differentiates GCRO from GCT remains the existence of eosinophilic, atypical osteoids surrounded by an osteoblastic rim. Additionally, invasive permeative infiltration, nuclear pleomorphism, atypical mononuclear spindle cells with anaplasia and the formation of irregularly marginated eosinophilic osteoid are the key sarcomatous features of GCRO that are unlikely to be present in GCT.^18,19,20^ Furthermore, MDM2 and CDK4 are amplified in low-grade osteosarcoma, as demonstrated by recent studies. This may also help differentiate GCT from GCRO. A high Ki67 proliferative index of more than 20% in GCRO has been shown to be helpful in distinguishing it from GCT.^20^ Therefore, it is crucial to obtain a sufficient amount of tissue sample for histopathology to avoid sampling bias in order to correctly distinguish between GCT and GCRO.^21^

The course of treatment for GCRO is identical to that for traditional osteosarcoma.^22^ It involves radical surgical resection, followed by chemotherapy and radiation. With distinct surgical margins, the survival rate can be as high as 80%. When positive surgical margins are present, radiotherapy is used as an adjuvant treatment before chemotherapy is started.^2^ Nonetheless, there is minimal difference in the survival rate between GCRO and high-grade osteosarcoma.^20^

## Conclusion

In this case, the suspected diagnosis based on clinical findings and radiological investigations was of osteosarcoma and malignant GCT. The diagnosis of giant cell-rich osteosarcoma was confirmed on biopsy, an extremely rare variant that may be easily misdiagnosed as a malignant GCT of the spine, both radiologically and histologically. However, the presence of irregular enhancing soft tissue components and blood-fluid levels on MRI may represent aggressive giant cell containing tumour like osteosarcoma and radiological suspicion could be raised. Finally, the presence of eosinophilic atypical osteoids surrounded by an osteoblastic rim and amplification of MDM2 or CDK4 genes can help distinguish GCRO from malignant GCT.
